# Uncontrolled Diabetes Mellitus Has No Major Influence on the Platelet Transcriptome

**DOI:** 10.1155/2018/8989252

**Published:** 2018-11-01

**Authors:** Thomas G. Nührenberg, Marco Cederqvist, Federico Marini, Christian Stratz, Björn A. Grüning, Dietmar Trenk, Harald Binder, Ralf Gilsbach, Franz-Josef Neumann, Lutz Hein

**Affiliations:** ^1^Institut für Experimentelle und Klinische Pharmakologie und Toxikologie, Albert-Ludwigs-Universität Freiburg, D-79104 Freiburg, Germany; ^2^Universitäts-Herzzentrum Freiburg, Bad Krozingen, Abteilung für Kardiologie und Angiologie II, D-79189 Bad Krozingen, Germany; ^3^Institut für Medizinische Biometrie, Epidemiologie und Informatik, Universitätsmedizin der Johannes-Gutenberg-Universität Mainz, D-55101 Mainz, Germany; ^4^Institut für Informatik, Albert-Ludwigs-Universität Freiburg, D-79110 Freiburg, Germany; ^5^Institut für Medizinische Biometrie und Statistik (IMBI), Albert-Ludwigs-Universität Freiburg, D-79104 Freiburg, Germany; ^6^BIOSS Centre for Biological Signaling Studies, University of Freiburg, D-79104 Freiburg, Germany

## Abstract

**Background:**

Diabetes mellitus (DM) has been associated with increased platelet reactivity as well as increased levels of platelet RNAs in plasma. Here, we sought to evaluate whether the platelet transcriptome is altered in the presence of uncontrolled DM.

**Methods:**

Next-generation sequencing (NGS) was performed on platelet RNA for 5 patients with uncontrolled DM (HbA1c 9.0%) and 5 control patients (HbA1c 5.5%) with otherwise similar clinical characteristics. RNA was isolated from leucocyte-depleted platelet-rich plasma. Libraries of platelet RNAs were created separately for long RNAs after ribosomal depletion and for small RNAs from total RNA, followed by next-generation sequencing.

**Results:**

Platelets in both groups demonstrated RNA expression profiles characterized by absence of leukocyte-specific transcripts, high expression of well-known platelet transcripts, and in total 6,343 consistently detectable transcripts. Extensive statistical bioinformatic analysis yielded 12 genes with consistently differential expression at a lenient FDR < 0.1, thereof 8 protein-coding genes and 2 genes with known expression in platelets (*MACF1* and* ITGB3BP)*. Three of the four differentially expressed noncoding genes were YRNAs (*RNY1, RNY3, *and* RNY4*) which were all downregulated in DM. 23 miRNAs were differentially expressed between the two groups. Of the 13 miRNAs with decreased expression in the diabetic group, 8 belonged to the DLK1–DIO3 gene region on chromosome 14q32.2.

**Conclusions:**

In this study, uncontrolled DM had a remote impact on different components of the platelet transcriptome. Increased expression of* MACF1*, together with supporting predicted mRNA-miRNA interactions as well as reduced expression of RNYs in platelets, may reflect subclinical platelet activation in uncontrolled DM.

## 1. Introduction

Patients with diabetes mellitus are at high risk for cardiovascular events such as myocardial infarction or stroke. Blood platelets play a pivotal role in hemostasis and thrombus generation and have been shown to be hyperreactive in patients with diabetes mellitus [1, 2]. Increased calpain activation has been identified as one mechanism leading to enhanced secretion of platelet *α*-granule proteins [3] which in turn might promote atherosclerosis in diabetic patients. Aside from various cytokines stored in *α*-granules as well as small molecules such as ATP, ADP, and serotonin in dense granules, platelets contain abundant microRNAs or miRNAs [4, 5]. These short, noncoding RNAs are under intense clinical and experimental investigation due to their potential as biomarkers and as intercellular messengers [1]. Platelet miRNAs and mRNAs can be secreted within vesicles [2–4] and lead to changes in gene expression of endothelial cells.

With regard to diabetes mellitus, Zampetaki et al. reported an unexplained loss of several endothelial- and platelet-derived miRNAs in the plasma of diabetic patients [5]. On the other hand, more recent data from the same group clearly indicate that levels of plasma miRNAs largely correlate with platelet miRNAs and in part with platelet activation levels [6, 7]. Disease-specific differences in plasma miRNAs should therefore either be due to altered release from platelets or altered clearance from the plasmatic compartment. Assessing the former possibility, our group has analyzed platelet miRNAs in a cohort of 60 patients with diabetes mellitus [8]. Compared to nondiabetic patients, patients with well-controlled disease had no major changes in the platelet miRNA profile. However, potential differences mediated by the presence of diabetes mellitus might have been attenuated by the overall good glucose control in the diabetic patients.

We therefore sought to further investigate the RNA profile of platelets from patients with uncontrolled diabetes mellitus in comparison to matched control patients without diabetes mellitus. In the current study, we used next-generation sequencing of both long and short RNAs, followed by comprehensive bioinformatic analysis.

## 2. Material and Methods

### 2.1. Subjects, Exclusion Criteria, and Blood Sampling

Blood was drawn from 10 patients that were electively hospitalized at the Universitäts-Herzzentrum Freiburg • Bad Krozingen and in stable clinical condition. Patients with unstable angina pectoris, myocardial infarction with or without ST-elevation, significant renal dysfunction, malignancy and pathological platelet count were excluded. Patients with uncontrolled diabetes were required to have an HbA1c of 8.5% or greater at the time of blood sampling. For the control group, patients with similar clinical characteristics but absence of diabetes were selected. For platelet isolation, blood was collected into 7.5 ml trisodium citrate (0.106 mol / l) monovettes (Sarstedt, Nümbrecht, Germany) after peripheral venous puncture with a safety Multifly-Set® (Sarstedt, Nümbrecht, Germany) and immediately processed.

All participants gave written informed consent to participate in the study which was approved by the Ethics Committee of the Albert-Ludwigs-Universität Freiburg. The investigation conformed to the principles outlined in the Declaration of Helsinki.

### 2.2. Platelet Function Tests

Light transmission aggregometry (Platelet Aggregation Profiler PAP-4 Bio/Data Corporation Horsham, PA, USA) using adenosine diphosphate (ADP 5*μ*M and 20*μ*M), Thrombin Receptor Activating Peptide (TRAP 25 *μ*M), arachidonic acid (500mg/l), and collagen (1mg/l and 2.5 mg/l, collagen fibrils from equine tendo) as platelet activators was carried out on platelet-rich plasma (PRP) to analyse basic functional platelet properties and to evaluate the platelet reaction to acetylsalicylic acid. Furthermore, multiplate electric impedance aggregometry was performed in whole blood collected into 2.7 mL tubes containing r-hirudin (45*μ*g/mL, Sarstedt, Nümbrecht, Germany) using the Multiplate Analyzer® (Roche Diagnostics, Mannheim, Germany). TRAP, ASPI, ADP, and hsADP tests were performed according to manufacturers' instructions: recording the area under the curve of aggregation units over 6 minutes. Results are reported as aggregation units *∗* minute (AU *∗* min).

### 2.3. Purification of Platelets by Leukocyte Depletion and RNA Isolation

Platelet purification and leukocyte depletion were performed as previously described [8, 9]. In brief, platelet rich plasma (PRP) was obtained by centrifugation (Megafuge 1.0 RS; Heraeus, Hanau, Germany) of citrated blood samples at 2040 rpm (750 rcf) for 2 min without brake. Depletion of leukocytes was achieved by negative selection using CD45^+^ magnetic beads (EasySep™; STEMCELL Technologies, Köln, Germany) according to the manufacturer's instructions. Total RNA including miRNA was extracted from the leukocyte depleted platelet pellets using the miRNeasy Mini Kit (Qiagen GmbH, Hilden, Germany) according to the manufacturer's recommendations. The eluted RNA was stored at −80°C.

### 2.4. Library Preparation and Next-Generation Sequencing

RNA yield was determined using an Agilent 2100 bioanalyzer with the eukaryote total RNA pico assay (Agilent, Waldbronn, Germany). In average 26.8 ± 7.4 ng total RNA were subjected to depletion of ribosomal RNA using the RiboMinus™ Eukaryote System v2 (life technologies, Darmstadt, Germany) according to manufacturers' instructions and directly used for preparation of 10 libraries with the NEBNext® Ultra™ Directional RNA Library Prep Kit for Illumina® (New England BioLabs Inc., Frankfurt am Main, Germany). Library size was assessed before sequencing using the Agilent 2100 bioanalyzer high sensitivity DNA analysis kit. In total, 6 small RNA libraries were successfully created from in average 84.4 ± 19.1 ng of total RNA with NEBNext® Small RNA Library Prep Set for Illumina® (New England BioLabs Inc., Frankfurt am Main, Germany). Barcoded libraries were sequenced on an Illumina HiSeq 2500.

### 2.5. Statistical Analysis and Bioinformatic Analysis

All sequencing data have been deposited in the European nucleotide archive (ENA; http://www.ebi.ac.uk/ena) under the accession number PRJEB18466. The datasets were independently analyzed by TN and FM at the respective locations. Both datasets were aligned to the hg38 version of the human genome, either by the Tophat2 (TN) [10] or by STAR aligner (FM) [11]. Data were analyzed for different gene expression with HTSeq [12] and DESeq2 [13] either without further data reduction or after removal of putative PCR duplicates by picard [https://github.com/broadinstitute/picard] or SAMtools, respectively [14]. Genes were considered differentially expressed at an FDR of <0.1 if they were identified by at least two of the three analysis conditions. This avoids artifacts from specific analysis pipelines, while at the same time the lenient level of 0.1 reflects the idea that potential differences should not be missed. Datasets for small RNA-Seq were analyzed similarly, but without duplicate removal. In addition, both datasets were analyzed in a similar fashion with more recent tools for detection of differential gene expression such as edgeR incorporating a quasilikelihood approach (edgeR-QLF) [15] and RNentropy [16] to further evaluate the robustness of results. To account for factors that might affect gene expression aside from Diabetes mellitus, we evaluated the effects of clinical characteristics on the principal components of gene expression, in such a way that important characteristics could subsequently be entered as covariates to the design of the experiment in the generalized linear model framework provided by DESeq2. If not indicated otherwise, clinical data are presented with median and interquartile range in brackets.

### 2.6. MiRNA-mRNA Interactions and Target Prediction

Differentially expressed miRNAs and mRNAs were analysed by Magia [2], a web tool for integrated analysis of mRNAs and miRNAs [17]. In detail, the prediction tools Targetscan [18], PicTar [19], and RNA22 [20] were used in complementary fashion.

## 3. Results

### 3.1. Baseline Characteristics

The 5 diabetic and 5 nondiabetic patients were well matched with regard to clinical and laboratory parameters as well as medication (Table 1). Concerning HbA1c, diabetic patients showed a median of 9.0%  [8.5-9.9%] compared to 5.5%  [5.4-5.7%] in the control group as intended. C-reactive protein was also significantly increased in diabetic patients (0.8 [0.6-1.2] mg/dl versus 0.3 [0.1-0.3] mg/dl), with values slightly above normal reference values (Table 1).

### 3.2. Multiplate Electric Impedance and Light Transmission Aggregometry

Multiplate electric impedance aggregometry with routine stimulation protocols showed no major differences in aggregation characteristics between the two groups. Light transmission aggregometry revealed decreased aggregation in diabetic patients upon low dose collagen stimulation and a trend to less aggregation upon stimulation with TRAP (Table 2).

### 3.3. Platelet Purification and Leukocyte Depletion

**Figure 1:**
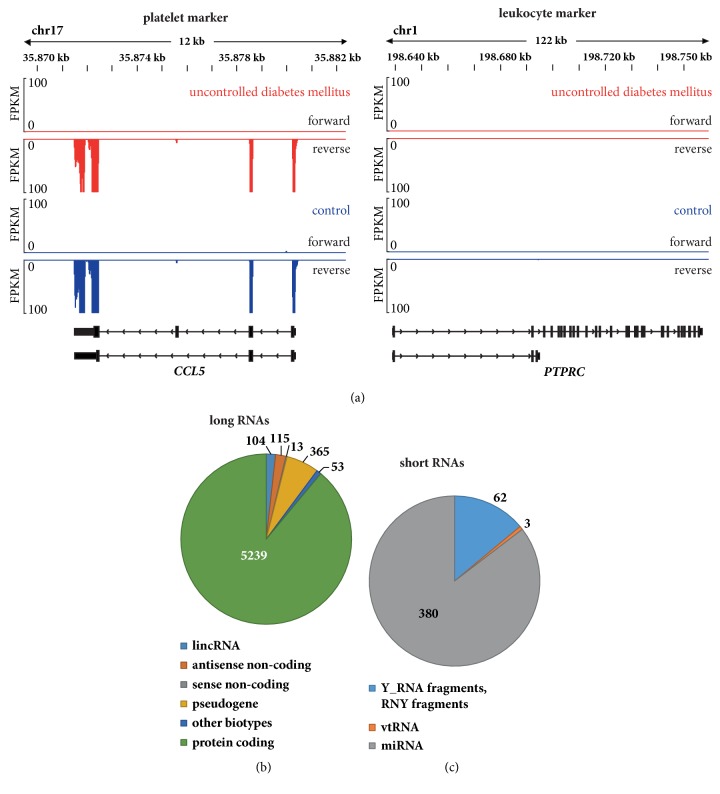
**Purity and gene biotypes of platelet RNAs**. (a) Genome browser view for transcripts of the platelet-enriched gene* CCL5* (left panel) and the leukocyte-specific gene* PTPRC* (CD45, right panel). FPKM denotes fragments per kilobase of transcript per million mapped reads. (b) Gene biotypes for mapped long RNAs. (c) Gene biotypes for mapped short RNAs.

Analysis of platelet and leukocyte marker transcripts confirmed the high degree of purity of the platelet preparations. The chemokine* CCL5* was highly expressed in platelets (Figure 1(a), left panels), whereas leukocyte markers, including CD45 (*PTPRC*), were not detectable (Figure 1(a), right panels).

### 3.4. Detection of Platelet Transcripts by Long RNA Sequencing and Influence of Uncontrolled Diabetes Mellitus

**Figure 2:**
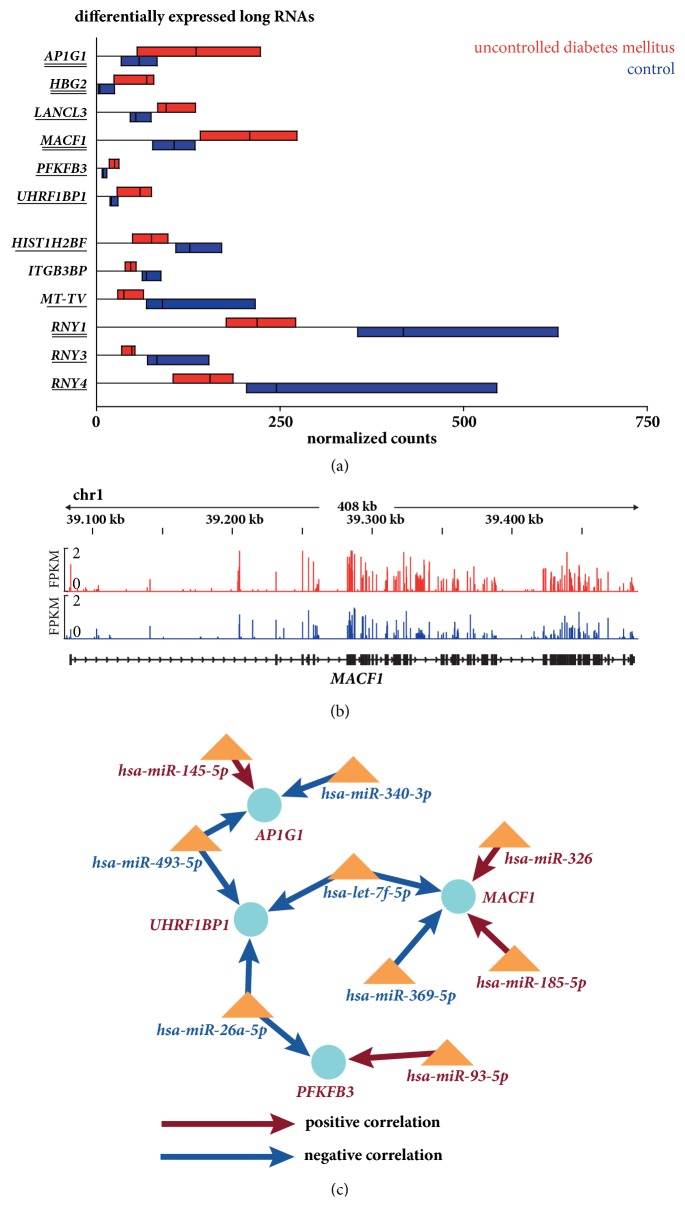
**Differential long RNA expression and mRNA-miRNA interaction network**. (a) Visualization of differentially expressed long RNAs by floating bars depicting the range of normalized counts from minimal to maximal values within in the respective groups. Black lines within the boxes represent the median. Red boxes show counts for transcripts of patients with uncontrolled diabetes mellitus, blue boxes show values of control patients. Underlined genes were also differentially expressed by analysis with edgeR-QLF, either after duplicate removal with picard (single line) or also with SAMtools (double line). (b) Genome browser view for transcripts of the* MACF1* gene. Transcripts of patients with uncontrolled diabetes mellitus are shown in red, transcripts of control patients in blue. FPKM denotes fragments per kilobase of transcript per million mapped reads. (c) Visualization of predicted mRNA-miRNA interactions obtained by analysis with Magia [2]. mRNAs are depicted by blue circles, miRNAs by orange triangles. Upregulation of mRNAs or miRNAs in uncontrolled diabetes mellitus is shown in red, downregulation in blue. The predicted direction of interaction is represented by the color of the arrows.

Mapping of sequenced ribosome-depleted RNA libraries with Tophat2 resulted in a median of 35.8 [32.7-39.0] million uniquely mapped reads. 5898 unique transcripts were consistently expressed in all samples. Of these, 5239 were protein-coding genes, 365 pseudogenes and 104 lincRNAs (Figure 1(b)). Regarding differential expression in diabetes mellitus, only 12 transcripts were detected as differentially expressed. Of these genes, 8 were protein-coding genes whereas 4 transcripts were noncoding RNAs (Table 3, Figure 2(a)). Adjustment for several clinical variables according to principal component analyses did not yield consistent effects of clinical characteristics on gene expression. Therefore, no adjustment was performed. Additional analysis with RNentropy did not yield any differentially expressed gene while comparison of DESeq2 with edgeR-QLF resulted in a large overlap of differentially expressed genes (Table 3, Figure 2(a), Supplementary table 1).

### 3.5. Differences in Short Platelet RNA Sequencing in Diabetic versus Non-Diabetic Patients

In the short RNA sequencing data, 445 RNAs were consistently expressed in all samples. Of these, 380 belonged to miRNAs, 62 to fragmented YRNAs, and RNYs and 3 to vault RNAs (Figure 1(c)). 24 short RNAs thereof 23 miRNAs were differentially expressed between diabetic patients and controls. Of the 13 miRNAs with decreased expression in the diabetic group, 8 belonged to the DLK1–DIO3 gene region on chromosome 14q32.2 (Table 3). Additional analysis with edgeR-QLF identified differentially expressed miRNAs, with complete overlap in miRNAs with decreased expression in the diabetic group and DLK1–DIO3 gene region (Table 3, Supplementary table 2).

### 3.6. Differentially Expressed miRNAs and mRNAs form a Putative Regulatory Network

miRNA target analysis by Magia [2] revealed a network including 4 of the 8 differentially expressed protein-coding genes. In particular, 4 of the 6 protein-coding genes with increased expression in diabetes mellitus belonged to the network. All 12 predicted positive or negative miRNA correlations were in line with the observed increased expression of the 4 protein-coding genes (Figure 2(b)).

## 4. Discussion

As main finding, no major differences are found in small and long RNAs in platelets of diabetic patients with poor glycemic control, compared to those from nondiabetic controls with similar clinical characteristics.

Few studies have measured the expression of platelet miRNA in diabetic patients and non-diabetic controls [8, 21]. Elgheznawy et al. reported a reduced expression of 4 miRNAs in platelets from eight diabetic patients compared to age-matched healthy controls [21]. In this study, no leukocyte-depletion of platelets was performed. This is relevant since miRNA expression was normalized to 18S ribosomal RNA which is highly expressed in nucleated cells such as leukocytes. In this context, we and others have shown that diabetic patients exhibit higher leukocyte counts [8, 22] than nondiabetic controls. Therefore, it cannot be excluded that higher leukocyte contamination explains the described differences. Another group has recently reported moderate reductions of 4 platelet miRNAs from diabetic patients [23]. In this study, qPCR assays were normalized to a small nucleolar RNA that could also easily be influenced by very low amounts of contaminating leukocytes [23].

We have previously reported the absence of major changes in the platelet miRNA profile in a well-matched cohort of sixty patients when leukocyte-depletion and array-technology was applied [8]. The current data, again with proof of leukocyte depletion, extend these findings to the entire transcriptome in individuals with poor glycemic control.

The few specific differences found in mRNA and miRNA expression yielded a consistent regulatory network, which is highly suggestive of persistent miRNA-mRNA target interactions in platelets. The observed increased expression of* MACF1* might be relevant in the context of platelet activation.* MACF1* is involved in the cross-linking of microtubules and actin filaments [24], structural components important for maintenance of the discoid platelet shape [25, 26], and granule secretion [27]. Furthermore, reduced expression of* ITGB3BP*, also known as *β*3-endonexin, might prevent platelet aggregation since* ITGB3BP *increases the affinity of integrin *α*_IIb_*β*_3_ for fibrinogen binding [28]. Yet, the relevance of these observations for platelet function is currently unknown. It is well possible that the observed changes are only a reminiscence of changes that are already present in the megakaryocyte. As platelets lack genomic DNA, two of the differentially expressed genes,* UHRF1BP1* and* HIST1H2BF*, may indeed reflect transcriptome differences between diabetic and non-diabetic megakaryocytes.* HIST1H2BF* encodes for a histone H2B variant, which represents an essential chromatin component. UHRF1BP1 interacts with DNA methyltransferases and may thus modulate DNA methylation, gene expression, and cellular differentiation.

A recent report has shown that translation in platelet is confined to the first hours of the platelet life span [29]. One could therefore speculate that individuals with higher platelet turnover dispose of platelets that are more adaptive to pathologic activation. Yet, the presence of higher platelet turnover in patients with diabetes mellitus remains to be shown. Regarding platelet function testing, no difference between diabetic and nondiabetic patients could be observed. Given the correlation of RNY fragments in plasma originating from platelets and platelet reactivity, RNY in plasma might be a more sensitive marker of platelet activation than classic platelet function tests [6]. This notion is underlined by our finding of reduced RNY expression in platelets from patients with uncontrolled diabetes mellitus, suggestive of increased secretion of RNY into plasma. Noteworthy, a substantial proportion of downregulated miRNAs belonged to the DLK1–DIO3 gene region on chromosome 14q32.2. Edelstein et al. showed that black Americans with increased PAR4-mediated platelet reactivity have also decreased expression of miRNAs within the DLK1–DIO3 gene region [30]. Similarly, miRNAs of this gene region were epigenetically repressed in islet cells from diabetic patients [31]. It therefore appears that downregulation of these miRNAs may be a general feature of type II diabetes but may indirectly influence platelet reactivity.

### 4.1. Limitations

One limitation of the present study is its small sample size. However, use of next-generation sequencing allowing precise determination of genome-wide presence of short and long transcripts precludes selection or normalization bias that is inherent in other array- or qPCR-based techniques. Certainly, minor differences could have been substantiated by enlarging the sample size. Yet, we already chose a lenient level FDR, to counter potential lack of power due to sample size. In addition, we employed different tools for detection of differential gene expression which may help to reduce the number of false positives. The identified set of differentially expressed genes in diabetic platelets will be an important basis for validation in a larger cohort.

Recently, the presence of circular RNAs in platelets was reported pointing to a degraded transcriptome [32]. An analysis using PTESFinder [33] with regard to differences in diabetic patients was not meaningful due to the sequencing length of 50 bp in our study (as compared to at least 75 bp recommended sequencing length for computational identification of circRNAs). In addition, we did not detect an increased platelet reactivity in diabetic patients using the Multiplate impedance aggregometry which is recommended by current guidelines for platelet function testing [34]. Flow cytometric analysis of monocyte-platelet aggregates assessing low-level platelet activation may have established subclinical platelet activation in diabetic patients.

As another limitation, we did not include a control group of patients with well-controlled diabetes. Hence, we cannot differentiate whether the observed changes depend on glycemic control or are driven by other factors present in diabetic patients. Yet, we did not see pronounced changes in miRNA levels of patients with controlled diabetes as compared to patients without diabetes previously [8].

### 4.2. Conclusions

In summary, uncontrolled diabetes is associated with minor differences in short and long platelet RNAs. Increased expression of* MACF1*, together with supporting predicted mRNA-miRNA interactions as well as reduced expression of RNYs in platelets, may reflect subclinical platelet activation in uncontrolled diabetes mellitus.

## Figures and Tables

**Table 1:** Baseline characteristics of the patients. BMI: body mass index and ASA: acetylsalicylic acid.

	**Diabetes **	**no Diabetes**	**p value**
Male sex	3 (60%)	3 (60%)	1
Age (years)	70 [63-72]	63 [59-70]	0.492
BMI (kg/m^2^)	33.4 [32.0-35.3]	31.8 [30.5-38.8]	0.841
Hypertension	5 (100%)	4 (80%)	1
Active smoking	1 (20%)	1 (20%)	1
Coronary artery disease	4 (80%)	2 (40%)	0.524
Remote coronary intervention	3 (60%)	2 (40%)	1
Reduced left ventricular function	2 (40%)	2 (40%)	1
**Medication**			
Insulin	3 (60%)	0 (0%)	0.167
Oral antidiabetics	3 (60%)	0 (0%)	0.167
ASA	3 (60%)	3 (60%)	1
Oral anticoagulation	3 (60%)	2 (40%)	1
Statins	3 (60%)	2 (40%)	1
**Laboratory parameters**			
Platelets (10^3^/*μ*l)	236 [183-238]	212 [207-225]	1
Leukocytes (10^3^/*μ*l)	9.0 [6.3-9.2]	8.6 [6.2-9.0]	0.548
Hemoglobin (g/dl)	13.4 [12.6-13.8]	14.7 [14.3-14.8]	0.143
Total cholesterol (mg/dl)	163 [160-168]	178 [168-219]	0.111
LDL cholesterol (mg/dl)	104 [65-106]	126 [114-128]	0.056
HDL cholesterol (mg/dl)	38 [29-41]	49 [42-87]	0.111
Triglycerides (mg/dl)	203 [100-280]	150 [118-151]	0.690
Serum glucose (mg/dl)	255 [202-256]	97 [87-107]	0.151
HbA1c (%)	9.0 [8.5-9.9]	5.5 [5.4-5.7]	0.008
C-reactive protein (mg/dl)	0.8 [0.6-1.2]	0.3 [0.1-0.3]	0.048
Creatinine (mg/dl)	1.0 [0.9-1.5]	0.9 [0.8-1.0]	0.444

**Table 2:** Multiplate electric impedance and light transmission aggregometry. TRAP: thrombin receptor-activating peptide, ADP: adenosine-diphosphate, HS: high-sensitivity, and AU: area under the curve.

	**Diabetes **	**no Diabetes**	**p**
			**(Mann-Whitney-U-test)**
**Multiplate electric impedance aggregometry**			
TRAPtest (AU*∗*min)	748 [721-1241]	941 [924-1019]	0.690
ADPtest (AU*∗*min)	707 [520-741]	696 [692-718]	0.841
ADPtest HS (AU*∗*min)	607 [400-610]	509 [461-568]	1
ASPItest (AU*∗*min)	342 [188-346]	413 [255-599]	0.518
**Light transmission aggregometry**			
TRAP 25*μ*M (% aggregation)	85 [84-86]	100 [100-100]	0.087
ADP 5*μ*M (% aggregation)	47 [34-49]	66 [44-72]	0.310
ADP 20*μ*M (% aggregation)	82 [67-84]	88 [74-94]	0.548
Arachidonic acid 500mg/l (% aggregation)	7 [7-10]	9 [6-96]	0.802
Collagen 1mg/l (% aggregation)	10 [10-11]	18 [14-74]	0.016
Collagen 2.5mg/l (% aggregation)	28 [23-43]	60 [39-100]	0.103

**Table 3:** Differential short RNA expression in platelets from patients with uncontrolled diabetes mellitus. + marks miRNAs that belong to the DLK1–DIO3 gene region on chromosome 14q32.2. FDR: Bonferroni-Hochberg-adjusted False-Discovery-Rate. miRNAs in bold were also differentially expressed by analysis with edgeR-QLF.

	**gene biotype**	**log**_**2**_ ** fold change**	**FDR**
		(Diabetes vs. Control)	
*hsa-miR-106b-3p*	miRNA	0.91	0.07
***hsa-miR-143-5p***	**miRNA**	**1.73**	**0.06**
***hsa-miR-145-5p***	**miRNA**	**1.90**	**0.06**
*hsa-miR-152-3p*	miRNA	0.75	0.07
*hsa-miR-185-5p*	miRNA	0.84	0.07
*hsa-miR-326*	miRNA	1.06	0.08
*hsa-miR-425-3p*	miRNA	0.73	0.09
*hsa-miR-652-3p*	miRNA	0.85	0.01
*hsa-miR-671-5p*	miRNA	1.27	0.07
*hsa-miR-93-5p*	miRNA	0.77	0.06

*hsa-let-7f-5p*	miRNA	-0.79	0.09
***hsa-miR-128-3p***	**miRNA**	**-1.65**	**0.00**
***hsa-miR-26a-5p***	**miRNA**	**-1.18**	**0.07**
***hsa-miR-340-3p***	**miRNA**	**-1.45**	**0.03**
+*** hsa-miR-493-5p***	**miRNA**	**-1.25**	**0.07**
+*** hsa-miR-411-3p***	**miRNA**	**-1.31**	**0.06**
+*** hsa-miR-543***	**miRNA**	**-1.45**	**0.06**
+*** hsa-miR-495-3p***	**miRNA**	**-1.06**	**0.08**
+*** hsa-miR-654-3p***	**miRNA**	**-1.08**	**0.08**
+*** hsa-miR-539-3p***	**miRNA**	**-2.18**	**0.02**
+*** hsa-miR-485-3p***	**miRNA**	**-1.17**	**0.07**
+*** hsa-miR-369-5p***	**miRNA**	**-1.55**	**0.00**
*Y_RNA.518-20-3p*	misc RNA	-1.77	0.07

## Data Availability

All sequencing data have been deposited in the European nucleotide archive (ENA; http://www.ebi.ac.uk/ena) and is available under the accession number PRJEB18466.

## Abbreviations

MiRNA: MicroRNA
PRP: Platelet-rich plasma
RNA-Seq: RNA-Sequencing.

## Supplementary Materials

Supplementary Materials Supplementary table 1: Differentially expressed long RNAs by edgeR-QLF. Supplementary table 2: Differentially expressed short RNAs by edgeR-QLF.
