# Supplementary material for: Uncontrolled Diabetes Mellitus Has No Major Influence on the Platelet Transcriptome
Source: Biomed Res Int. 2018 Nov 1;2018:8989252. doi: 10.1155/2018/8989252 (PMC6241365; doi:10.1155/2018/8989252)
Supplement: Supplementary Materials — Supplementary table 1: Differentially expressed long RNAs by edgeR-QLF. Supplementary table 2: Differentially expressed short RNAs by edgeR-QLF. [file 8989252.f1.docx]

| **Supplementary table 1: Differentially expressed long RNAs by edgeR-QLF** | | | |
| --- | --- | --- | --- |
|  |  |  |  |

| **Ensembl gene ID** | **Gene symbol** | **logFC** | **logCPM** | **F** | **P-Value** | **FDR** |
| --- | --- | --- | --- | --- | --- | --- |
|  |  |  |  |  |  |  |
| **Genes differentially expressed after duplicate removal with picard** | | | | |  |  |
| ENSG00000241781 | AL161626.1 | -3.669 | 2.245 | 18.200 | 2.0E-05 | 0.009 |
| ENSG00000164111 | ANXA5 | 1.938 | 3.715 | 20.187 | 7.1E-06 | 0.005 |
| ENSG00000166747 | AP1G1 | 1.268 | 6.722 | 30.134 | 4.3E-08 | 1.1E-04 |
| ENSG00000186318 | BACE1 | 1.990 | 3.232 | 15.300 | 9.2E-05 | 0.032 |
| ENSG00000146540 | C7orf50 | -1.468 | 4.493 | 18.862 | 1.4E-05 | 0.008 |
| ENSG00000121691 | CAT | 1.434 | 4.677 | 15.824 | 1.2E-04 | 0.038 |
| ENSG00000256263 | DDX11L8 | -3.188 | 2.970 | 27.816 | 1.3E-07 | 2.2E-04 |
| ENSG00000126767 | ELK1 | 2.214 | 2.702 | 12.737 | 3.6E-04 | 0.088 |
| ENSG00000227811 | FAM212B-AS1 | -1.079 | 6.313 | 18.736 | 1.9E-05 | 0.009 |
| ENSG00000163430 | FSTL1 | 0.838 | 8.465 | 18.720 | 1.5E-05 | 0.008 |
| ENSG00000243955 | GSTA1 | -2.525 | 2.947 | 19.029 | 1.3E-05 | 0.008 |
| ENSG00000196565 | HBG2 | 2.772 | 5.286 | 70.576 | 2.4E-15 | 3.3E-11 |
| ENSG00000277224 | HIST1H2BF | -0.949 | 6.865 | 17.932 | 2.3E-05 | 0.010 |
| ENSG00000158406 | HIST1H4H | -1.078 | 6.403 | 19.959 | 8.7E-06 | 0.006 |
| ENSG00000184678 | HIST2H2BE | -0.644 | 10.120 | 13.526 | 2.4E-04 | 0.062 |
| ENSG00000271361 | HTATSF1P2 | -2.281 | 3.140 | 18.008 | 2.2E-05 | 0.010 |
| ENSG00000115457 | IGFBP2 | 2.489 | 4.157 | 21.954 | 3.6E-05 | 0.015 |
| ENSG00000006459 | KDM7A | 0.964 | 7.120 | 19.635 | 9.4E-06 | 0.006 |
| ENSG00000147036 | LANCL3 | 0.895 | 6.466 | 14.260 | 1.6E-04 | 0.045 |
| ENSG00000090382 | LYZ | 1.666 | 3.602 | 14.015 | 1.9E-04 | 0.052 |
| ENSG00000127603 | MACF1 | 0.984 | 7.460 | 21.749 | 3.1E-06 | 0.003 |
| ENSG00000275110 | MIR6087 | -2.360 | 3.221 | 20.937 | 4.8E-06 | 0.004 |
| ENSG00000198727 | MT-CYB | -0.592 | 12.612 | 14.011 | 1.9E-04 | 0.052 |
| ENSG00000211459 | MT-RNR1 | -0.777 | 15.308 | 29.307 | 8.6E-08 | 1.7E-04 |
| ENSG00000210082 | MT-RNR2 | -0.628 | 17.003 | 19.250 | 2.1E-05 | 0.009 |
| ENSG00000210176 | MT-TH | -1.433 | 5.868 | 29.878 | 4.7E-08 | 1.1E-04 |
| ENSG00000210112 | MT-TM | -1.311 | 6.098 | 27.091 | 2.0E-07 | 2.7E-04 |
| ENSG00000210077 | MT-TV | -1.395 | 6.363 | 32.820 | 1.0E-08 | 3.5E-05 |
| ENSG00000197616 | MYH6 | -4.707 | 1.686 | 14.738 | 1.2E-04 | 0.039 |
| ENSG00000170113 | NIPA1 | 1.235 | 6.000 | 22.506 | 2.5E-06 | 0.002 |
| ENSG00000103512 | NOMO1 | -1.305 | 5.016 | 18.244 | 1.9E-05 | 0.009 |
| ENSG00000159339 | PADI4 | -0.991 | 6.313 | 16.981 | 3.8E-05 | 0.015 |
| ENSG00000277258 | PCGF2 | -2.395 | 2.569 | 13.256 | 2.8E-04 | 0.069 |
| ENSG00000141179 | PCTP | 1.245 | 4.452 | 13.454 | 2.4E-04 | 0.063 |
| ENSG00000170525 | PFKFB3 | 1.288 | 4.423 | 13.981 | 1.8E-04 | 0.052 |
| ENSG00000068878 | PSME4 | 0.973 | 6.708 | 18.134 | 2.1E-05 | 0.009 |
| ENSG00000120899 | PTK2B | 1.132 | 5.245 | 15.274 | 9.4E-05 | 0.032 |
| ENSG00000188672 | RHCE | 2.776 | 3.102 | 23.665 | 1.2E-06 | 0.001 |
| ENSG00000168159 | RNF187 | 1.630 | 3.977 | 16.583 | 5.5E-05 | 0.021 |
| ENSG00000173821 | RNF213 | 1.355 | 4.478 | 15.697 | 7.4E-05 | 0.027 |
| ENSG00000206892 | RNU6-42P | -1.329 | 5.102 | 19.773 | 8.7E-06 | 0.006 |
| ENSG00000201098 | RNY1 | -1.022 | 8.500 | 27.675 | 1.4E-07 | 2.2E-04 |
| ENSG00000202354 | RNY3 | -1.096 | 6.240 | 19.915 | 8.1E-06 | 0.006 |
| ENSG00000252316 | RNY4 | -1.001 | 7.927 | 24.267 | 8.4E-07 | 0.001 |
| ENSG00000274253 | RP11-566K19.6 | 3.346 | 2.385 | 16.262 | 5.6E-05 | 0.021 |
| ENSG00000234449 | RP11-706O15.3 | 2.112 | 3.444 | 15.979 | 9.7E-05 | 0.032 |
| ENSG00000170542 | SERPINB9 | 1.108 | 5.330 | 14.821 | 1.2E-04 | 0.038 |
| ENSG00000146409 | SLC18B1 | 1.887 | 3.263 | 14.346 | 1.5E-04 | 0.044 |
| ENSG00000197157 | SND1 | 1.548 | 4.028 | 16.427 | 5.3E-05 | 0.021 |
| ENSG00000135426 | TESPA1 | -2.304 | 4.225 | 24.785 | 5.0E-06 | 0.004 |
| ENSG00000100580 | TMED8 | 0.885 | 6.546 | 14.435 | 1.5E-04 | 0.043 |
| ENSG00000187653 | TMSB4XP8 | -1.232 | 7.941 | 36.297 | 1.8E-09 | 1.3E-05 |
| ENSG00000162722 | TRIM58 | 0.676 | 9.946 | 14.492 | 1.5E-04 | 0.043 |
| ENSG00000065060 | UHRF1BP1 | 1.319 | 5.496 | 22.329 | 2.3E-06 | 0.002 |
| ENSG00000199990 | VTRNA1-1 | -1.618 | 5.616 | 34.709 | 4.1E-09 | 1.9E-05 |
| ENSG00000252802 | Y_RNA | -3.790 | 1.903 | 13.429 | 2.5E-04 | 0.063 |
|  |  |  |  |  |  |  |
| **Genes differentially expressed after duplicate removal with SAMtools** | | | | | |  |
| ENSG00000229349 | ACTG1P9 | 5.698 | 2.961 | 19.418 | 1.1E-05 | 0.016 |
| ENSG00000185736 | ADARB2 | 6.982 | 4.127 | 48.869 | 2.2E-11 | 3.0E-07 |
| ENSG00000169877 | AHSP | 1.445 | 6.113 | 16.091 | 6.1E-05 | 0.054 |
| ENSG00000166747 | AP1G1 | 1.123 | 7.440 | 16.846 | 4.1E-05 | 0.044 |
| ENSG00000185515 | BRCC3 | -6.251 | 2.334 | 14.559 | 1.4E-04 | 0.069 |
| ENSG00000133742 | CA1 | 1.201 | 8.389 | 18.255 | 5.0E-05 | 0.050 |
| ENSG00000124795 | DEK | -0.930 | 7.948 | 13.384 | 2.5E-04 | 0.100 |
| ENSG00000108406 | DHX40 | 2.418 | 4.176 | 13.816 | 2.0E-04 | 0.091 |
| ENSG00000178852 | EFCAB13 | -0.782 | 10.109 | 14.893 | 1.1E-04 | 0.065 |
| ENSG00000144820 | GPR128 | 4.151 | 3.043 | 13.899 | 2.0E-04 | 0.091 |
| ENSG00000213934 | HBG1 | 6.693 | 2.727 | 19.547 | 9.8E-06 | 0.016 |
| ENSG00000196565 | HBG2 | 2.027 | 5.647 | 23.858 | 1.1E-06 | 0.005 |
| ENSG00000204010 | IFIT1B | 2.196 | 5.028 | 18.371 | 2.4E-05 | 0.033 |
| ENSG00000115457 | IGFBP2 | 3.398 | 4.497 | 30.192 | 4.2E-08 | 3.0E-04 |
| ENSG00000006459 | KDM7A | 1.239 | 7.495 | 20.715 | 5.3E-06 | 0.013 |
| ENSG00000249307 | LINC01088 | 1.671 | 5.862 | 17.359 | 3.8E-05 | 0.044 |
| ENSG00000127603 | MACF1 | 0.972 | 8.224 | 15.769 | 7.2E-05 | 0.059 |
| ENSG00000250927 | MESTP3 | -3.980 | 3.060 | 13.597 | 2.3E-04 | 0.094 |
| ENSG00000187193 | MT1X | 2.302 | 4.265 | 13.352 | 2.6E-04 | 0.100 |
| ENSG00000180530 | NRIP1 | -0.910 | 8.647 | 15.004 | 1.1E-04 | 0.065 |
| ENSG00000159339 | PADI4 | -1.087 | 7.188 | 14.535 | 1.4E-04 | 0.069 |
| ENSG00000188672 | RHCE | 3.857 | 3.645 | 19.865 | 8.3E-06 | 0.016 |
| ENSG00000168159 | RNF187 | 2.420 | 4.156 | 13.655 | 2.2E-04 | 0.094 |
| ENSG00000207334 | RNU6-12P | -1.123 | 8.242 | 21.002 | 4.6E-06 | 0.013 |
| ENSG00000206965 | RNU6-5P | -1.529 | 6.682 | 23.118 | 1.5E-06 | 0.005 |
| ENSG00000221676 | RNU6ATAC | -0.961 | 8.295 | 15.674 | 7.5E-05 | 0.059 |
| ENSG00000201098 | RNY1 | -0.916 | 8.430 | 14.666 | 1.3E-04 | 0.069 |
| ENSG00000214380 | RP11-457K10.2 | -4.963 | 2.810 | 15.062 | 1.0E-04 | 0.065 |
| ENSG00000235916 | RP13-444K19.1 | -6.465 | 2.472 | 16.289 | 5.5E-05 | 0.051 |
| ENSG00000112902 | SEMA5A | -3.897 | 3.253 | 14.993 | 1.1E-04 | 0.065 |
| ENSG00000070182 | SPTB | 0.872 | 8.430 | 13.330 | 2.6E-04 | 0.100 |
| ENSG00000144057 | ST6GAL2 | -1.262 | 6.512 | 14.954 | 1.1E-04 | 0.065 |
| ENSG00000135426 | TESPA1 | -2.298 | 4.674 | 15.711 | 9.0E-05 | 0.063 |
| ENSG00000228499 | TMSB10P1 | 6.333 | 2.398 | 15.395 | 8.7E-05 | 0.063 |
| ENSG00000205542 | TMSB4X | -0.687 | 12.937 | 17.696 | 2.6E-05 | 0.033 |
| ENSG00000162722 | TRIM58 | 0.771 | 10.088 | 14.278 | 1.6E-04 | 0.079 |
| ENSG00000104691 | UBXN8 | 6.136 | 2.236 | 13.680 | 2.2E-04 | 0.094 |

| **Supplementary table 2: Differentially expressed short RNAs by edgeR-QLF** | | | | | | | | | | |
| --- | --- | --- | --- | --- | --- | --- | --- | --- | --- | --- |
|  | | |  | | |  | | |  | |
| **Gene symbol** | **logFC** | | **logCPM** | **F** | | **P-Value** | **FDR** | |  |  |
|  |  | |  |  | |  |  | |  |  |
| hsa-miR-1260a | -1.515 | | 6.257 | 9.699 | | 0.002 | 0.065 | |  |  |
| hsa-miR-128-3p | 1.622 | | 12.548 | 28.326 | | 1.1E-07 | 8.4E-05 | |  |  |
| hsa-miR-130b-5p | 1.285 | | 9.260 | 10.112 | | 0.001 | 0.054 | |  |  |
| hsa-miR-136-3p | 1.660 | | 6.639 | 11.589 | | 0.001 | 0.029 | |  |  |
| hsa-miR-143-5p | -2.085 | | 5.716 | 16.648 | | 4.6E-05 | 0.004 | |  |  |
| hsa-miR-145-5p | -3.264 | | 3.944 | 16.177 | | 6.0E-05 | 0.005 | |  |  |
| hsa-miR-21-5p | 0.571 | | 17.276 | 9.032 | | 0.003 | 0.086 | |  |  |
| hsa-miR-26a-5p | 0.731 | | 16.546 | 12.779 | | 3.6E-04 | 0.018 | |  |  |
| hsa-miR-323b-3p | 1.358 | | 7.819 | 8.817 | | 0.003 | 0.089 | |  |  |
| hsa-miR-339-5p | 1.295 | | 8.774 | 9.353 | | 0.002 | 0.076 | |  |  |
| hsa-miR-340-3p | 1.854 | | 10.399 | 24.274 | | 8.8E-07 | 2.3E-04 | |  |  |
| hsa-miR-369-3p | 1.761 | | 8.989 | 17.584 | | 2.8E-05 | 0.003 | |  |  |
| hsa-miR-369-5p | 2.178 | | 8.338 | 22.953 | | 1.7E-06 | 3.2E-04 | |  |  |
| hsa-miR-411-3p | 2.212 | | 6.726 | 19.377 | | 1.1E-05 | 0.001 | |  |  |
| hsa-miR-411-5p | 1.370 | | 10.112 | 13.129 | | 3.0E-04 | 0.016 | |  |  |
| hsa-miR-485-3p | 1.784 | | 8.026 | 15.146 | | 1.0E-04 | 0.007 | |  |  |
| hsa-miR-493-3p | 1.169 | | 9.559 | 8.860 | | 0.003 | 0.089 | |  |  |
| hsa-miR-493-5p | 1.873 | | 8.914 | 19.427 | | 1.1E-05 | 0.001 | |  |  |
| hsa-miR-495-3p | 1.677 | | 7.982 | 13.422 | | 2.5E-04 | 0.015 | |  |  |
| hsa-miR-539-3p | 3.348 | | 4.232 | 14.147 | | 1.7E-04 | 0.011 | |  |  |
| hsa-miR-543 | 2.041 | | 9.764 | 26.354 | | 3.2E-07 | 1.2E-04 | |  |  |
| hsa-miR-625-3p | 1.373 | | 9.526 | 11.996 | | 0.001 | 0.026 | |  |  |
| hsa-miR-625-5p | 1.375 | | 9.315 | 11.618 | | 0.001 | 0.029 | |  |  |
| hsa-miR-654-3p | 1.860 | | 9.869 | 22.628 | | 2.1E-06 | 3.2E-04 | |  |  |
| hsa-miR-6843-3p | 1.773 | | 5.529 | 10.824 | | 0.001 | 0.039 | |  |  |
| hsa-miR-99a-5p | 1.134 | | 11.424 | 11.327 | | 0.001 | 0.031 | |  |  |
